# Transforming growth factor beta family expression at the bovine feto-maternal interface

**DOI:** 10.1186/1477-7827-8-120

**Published:** 2010-10-15

**Authors:** Kumiko Sugawara, Keiichiro Kizaki, Chandana B Herath, Yoshihisa Hasegawa, Kazuyoshi Hashizume

**Affiliations:** 1 Laboratory of Veterinary Physiology, Department of Veterinary Medicine, Iwate University, Morioka, 020-8550 Iwate, Japan; 2 Current address: Agricultural Mutual Relief Association Joint Association in Miyagi Prefecture, Osaki-shi, 989-6117 Miyagi, Japan; 3 Laboratory of Reproductive Endocrinology, Department of Developmental Biology, National Institute of Agrobiological Sciences, Tsukuba, Ibaraki, Japan; 4 Current address: Department of Medicine, The University of Melbourne, Austin Repatriation Hospital, Heidelberg Heights, Victoria 3081, Australia; 5 Kitasato University School of Veterinary Medicine, Towada, 034-8628 Aomori, Japan

## Abstract

**Background:**

Endometrial remodelling is necessary for implantation in all mammalian species. The TGF beta super-family plays a crucial role in this event in humans and mice. However, the role of TGF beta super-family members during implantation is still unclear in ruminants. In the present study, the spacio-temporal expression of TGF beta super-family members including activin was explored in bovine trophoblasts and endometrial tissue during the peri-implantation period in order to elucidate whether it is essential for promoting cell proliferation at the implantation site.

**Methods:**

Gene expression in the fetal membrane and endometrium of the gravid and non-gravid horn around Day 35 of gestation were analyzed with a custom-made oligo-microarray in cattle. The expression of activin and its related genes was also analyzed with quantitative RT-PCR. Activin-like activity in trophoblastic tissue and BT-1 cells was examined using a fibroblast cell proliferation test and Western blotting.

**Results:**

The expression of various TGF beta super-family related genes including activin was detected in trophoblasts and the endometrium in cattle. The most intensive activin expression was found in the gravid horn endometrium, and rather intense expression was detected in the non-gravid trophoblastic tissue. Extracts from the fetal membrane including trophoblasts and purified activin both stimulated fibroblast proliferation effectively, and activin was immunologically detected in BT-1 cells, which have trophoblastic features.

**Conclusions:**

Specific expression of the activin gene (gene name: inhibin beta A) was found in the gravid horn endometrium during peri-implantation. An activin-like molecule, which was derived from the endometrium and trophoblasts, stimulated the proliferation of fibroblast cells. These results suggested that as in other species, the activity of TGF beta super-family members including activin-like molecules plays a pivotal role in endometrial remodelling, which is an essential process in implantation and placentogenesis during the peri-implantation period in cattle.

## Background

Implantation involves a complex process and various kinds of factors, during which time the fetal-maternal interface develops into the placenta. Trophoblast cells and maternal endometrial cells adhere to each other and produce the feto-maternal interface, which represents the beginning of implantation and endometrial remodelling. However, the cells from the fetus and mother only fuse and develop into the placentome in a limited area, namely the caruncle (CAR) on the endometrium in cattle [1]. Stromal cells proliferate in the CAR during peri-implantation, which corresponds to the proliferative activity of trophoblasts. In cattle, the rapid proliferation and differentiation of the early embryo and/or the conceptus begins in the gravid horn, which contains the fetus, and the conceptus grows with morphological changes such as the development of spherical, ovoid, filamentous, and elongated membranes, the latter of which then spreads to the other side of the uterine horn (non-gravid horn) [2-5]. In particular, endometrial and fetal trophoblastic cells markedly proliferate at the CAR, where the placentome develops as the feto-maternal interface including the epithelium and stroma. Various molecules derived from trophoblastic and endometrial cells including cytokines, growth factors, and hormones [6] may play an important role at this site in cattle, and the fusion of trophoblast and endometrial epithelial cells starts around 20 days of gestation [7-9]. Although these phenomena have been morphologically confirmed, the detailed mechanisms behind them are still unclear in ruminants. Various growth factors, such as heparin-binding epidermal growth factor (HB-EGF) and transforming growth factors (TGFs), promote crucial events during the peri-implantation period in humans and mice [10-14]. These growth factors may affect the initiation of endometrial proliferation during implantation in these species.

TGFβ superfamily members including activin and some growth factors participate in this critical event in mice and humans [15-17] but little information is available about this process in ruminants. A microarray study suggested that activin (gene name: inhibin βA) coordinates endometrial remodelling in cattle [18]. In other species, there is evidence that activin is involved in endometrial remodelling [15]. Activin, which was originally identified as a peptide growth factor obtained from the ovarian follicular fluid [19], is a disulphide-linked dimeric glycoprotein composed of two subunits in various combinations, βA and βA (homodimer), βA and βB (heterodimer), and βB and βB (homodimer), which were named activin A (gene symbol: INHβA), activin AB (INHβAB), and activin B (INHβB), respectively. Both the βA and βB subunits can also be combined with the α-subunit to form inhibin-A (αβA) and -B (αβB), respectively [20]. Follistatin, which is related to activin and inhibin, and can be purified from ovarian follicular fluid, binds to activin with high affinity and neutralizes activin bioactivity [21]. Although the TGFβ super-family members regulate the cell proliferation in various kinds of cells via complex mechanisms, they may stimulate endometrial cell activity and function [22,23]. In humans, activin plays a stimulatory role in decidualization and endometrial cell invasion [12,24,25].

The aims of this study are to confirm the expression of TGFβ super-family members during gestation, and to explore the factors that promote cell proliferation at the implantation site and the possible role of activin, which is a TGFβ super-family member, in trophoblasts and the endometrium during the peri-implantation period.

## Methods

### Tissue Preparation

The following tissues were used to obtain total RNA from Japanese Black cows: the fetal membrane (FM) and endometrium (END) of the gravid horn (GH) and/or the non-gravid horn (NGH) were collected separately. Some tissues such as the FM were only collected for microarray analysis, in which it was difficult to identify the cotyledonary (COT) and intercotyledonary (ICOT) sections during the early period of implantation. After day 50 of gestation COT, ICOT, caruncular (CAR) and intercarunclar (ICAR) endometrium were separately collected. Endometrial tissues for gene expression analysis were collected from cows on day 7-14 of the estrous cycle (n = 3). The main tissue collection schedules are summarized in Table 1. The day of artificial insemination was designated as Day 0 of gestation. All tissues used for the gene, biological, and biochemical analyses were snap frozen in liquid nitrogen and stored at -80°C until RNA and protein extraction. All animal experiments and procedures were approved by the Animal Experimental Committee of Iwate University and National Institute of Agrobiological Sciences.

**Table 1:** Tissue collection schedule for gene expression analysis

	Cyclic	GH	NGH	GH	NGH
**a: For microarray and quantitative RT -PCR (number of animals)**					
Day of gestation	END	FM	FM	END	END
35-37*	3	3	2	3	3
**b: For quantitative RT-PCR (number of animals)**					
Day of gestation	FM	COT	ICOT	CAR	ICAR
27-34	6	-	-	5	5
48-64		2	5	8	9
93-150		4	6	7	9
240-270		3	4	5	7

GH: gravid horn, NGH: non-gravid horn, FM: fetal membrane, END: endometrium, COT: cotyledonary fetal membrane, ICOT: intercotyledonary fetal membrane, CAR: caruncular tissue of the endometrium, ICAR: intercaruncular tissue of the endometrium. *:Data was shown in text and tables as Day 35.

**Figure 1:**
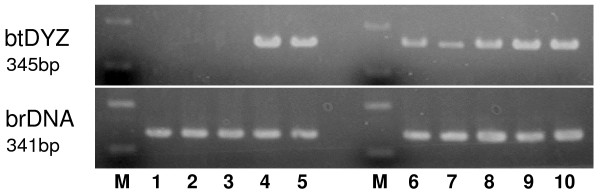
**Characteristics of fibroblastic cells (C cell) derived from trophoblastic tissue with male fetus**. Lane 1: stromal cells derived from mother endometrium, Lane 2: epithelial cells derived from mother epithelium, Lane 3: dermal cells from mother skin, Lane 4: stromal cells from trophoblastic tissue: Lane 5: stromal cells from fetus, Lane 6-10: fibroblastic cells (C cell) from trophoblastic tissue which has male fetus. These cells were established from different conceptus, respectively. btDYZ was amplified with RT-PCR using following male specific gene primers [28]. btDYZ, Forward: CCTTTAGGGGAAAATGGACTGACA, Reverse: ATGTGACACTGCTGGAAGG. brDNA (bovine ribosomal DNA, Control), Forward: CCAGTGCTCTGAATGTCAAA, Reverse: GCCTCCCAGTTATTCTACAC.

Bovine endometrial (E cells) or cotyledonary (C cells) fibroblast cells were derived from the endometrium and cotyledon, respectively, of Japanese Black cattle that were reared at the National Institute of Agrobiological Sciences [26,27] and used for the proliferation assay. In brief, small pieces of tissue were subjected to explant culture, and the cells that grew around the explanted tissue were collected and passaged at least three times to generate a fibroblast cell population, and then less than 10 passaged cells were used as fibroblast cells after immunohistochemical characterization with vimentin and cytokeratin, as described previously [27]. C cell was confirmed as a cell derived from cotyledonary tissue as shown in Figure 1 using Y-chromosome specific gene [28]. The bovine trophoblast cell line (BT-1), which was established in our laboratory [29], and bovine skin fibroblasts (SKIN) were prepared and used as a control in the total RNA expression and protein extraction analyses. Primary bovine SKIN cells were established from dermis tissue derived from the bovine shoulder. After all hair had been removed and the tissue had been rinsed in 70% ethanol, the tissue was washed in phosphate buffered saline (PBS) containing 100 IU/mL of penicillin and 100 μg/mL of streptomycin several times. It was then minced thoroughly with scissors and placed on plastic Petri dishes, before being carefully filled with DMEM/F-12 medium supplemented with 10% FBS. These cells were then cultured in Dulbecco's modified Eagle's medium/F-12 medium (DMEM/F-12, Sigma, Saint Louis, MI, USA) containing 100 IU/mL of penicillin and 100 μg/mL of streptomycin (Sigma), supplemented with 10% fetal bovine serum (FBS, HANA-NESCO, Tokyo, Japan), at 37°C in an atmosphere of 5% CO2. The confluent fibroblast cells were scale passaged using 0.25% trypsin-EDTA (Sigma) and plated on flasks. The BT-1 cells were cultured according to the previously described method [30].

### Protein extraction and purification

Frozen FM (Day 90 of gestation) was thawed in chilled PBS (containing penicillin and streptomycin) before being minced. After five minutes of PBS supplementation, they were centrifuged at 300 ×g for 10 minutes at 4°C, and the supernatant was removed. These steps were repeated until all blood cell factors had been removed from the supernatant. Following the addition of PBS, the minced tissues were homogenized using a polytron homogenizer (ULTRA TURRAX T25, IKA Works, NC, USA) and centrifuged at 44,000 ×g for 10 minutes at 4°C. The supernatant was then stored at -30°C until the proliferation assay.

The protein extraction from SKIN and BT-1 cells was performed using the following procedures: the SKIN cells were washed twice with serum-free medium (DMEM/F12) in close to confluent conditions and then incubated with medium (serum-free) at 37°C for two hours. After being washed twice, the cells were scraped using a cell scraper in PBS, and the resultant cell suspension was centrifuged at 1,000 ×g for 10 minutes at room temperature. The pellet was then re-suspended in two volumes of PBS, before being homogenized and centrifuged as in the FM procedure. The supernatant was used for protein analysis. The BT-1 cells were washed three times with serum-free medium and incubated in serum-free medium at 37°C for two hours to eliminate contaminating factors from the FBS. Then, all cells were cultured in 2 mL of serum-free medium for 48 h, collected, and used for protein analysis. The conditioned medium was collected after centrifugation at 3,000 ×g for 10 minutes. The pellet was then homogenized, and the supernatant was collected as mentioned for the SKIN cell procedure. To concentrate the conditioned medium, 2 mL of medium were mixed with 8 mL of cold acetone. After being incubated overnight at -30°C, the sample was centrifuged at 10,000 ×g for 10 minutes, and the pellet was suspended in 100 μL of PBS and used for further analysis.

An activin-like substance was extracted from the BT-1 cells using sulfated Cellulofine (Seikagaku biobusiness, Tokyo, Japan), which is an affinity chromatography reagent that concentrates TGFβ related molecules [31], according to the manufacturer's instructions. In brief, after the Cellulofine extract had been washed five times with PBS, 100 μg of BT-1 extract and 100 μL of Cellulofine were mixed and incubated for 2 hours at 4°C on the rotator. The mixtures were then washed three times with PBS, and 50 μg of PBS with 1 M NaCl were added. After 1 h incubation at 4°C, they were centrifuged at 10,000 ×g for 5 min at 4°C, and the supernatants were collected.

### Proliferation assay

The E and C cells were plated at 1 × 10^5 ^cells/dish and incubated in DMEM/F12 medium containing 10% FBS at 37°C in an atmosphere of 5% CO_2_. The cells were then washed twice with PBS and incubated in DMEM/F12 medium without FBS for 24 h at 37°C. The cells extracts (protein concentrations ranging from 1~100 μg/mL) and growth factors (as shown below) were diluted to each concentration with serum-free medium, and the cells were incubated with them for 48 h at 37°C. After the cells had been dissociated with 0.25% trypsin solution, suspended in medium, and mixed with 0.5% Trypan blue reagent (Wako Pure Chemical Industries, Osaka, Japan; 1:1), the total number of cells was counted using Thoma's hemocytometer. All assays were performed in triplicate. All values are presented as the mean ± SD.

Activin A, activin AB, and follistatin, which were purified by Y.H. [32], were used for cell proliferation analysis with 1~1000 ng/mL. Other growth factors were purchased from commercial companies: FGF2 (0.1~100 ng/mL, WAKO 067-02831), HB-EGF (0.1~100 ng/mL, WAKO 084-08281), and TGFβ1 (0.01~10 ng/mL, WAKO 204-14291) were from Wako chemical co.

### Microarray analysis

A customized bovine oligonucleotide microarray with 11,000 unique genes (GPL9136) was used to detect the genes expressed in the fetal membrane and endometrial tissues in the gravid and non-gravid uterine horn. The oligo-microarray produced by Agilent Technologies (Santa Clara, CA, USA) was used in this study. Sixty-mer nucleotide probes for the customized microarray were synthesized on a glass slide. The annotated bovine oligonucleotide array represented 10263 sequences, 4466 of which were known bovine genes, 5697 were unknown sequences and possible candidates for novel bovine genes, and 100 were internal references. All total RNA and mRNA tested including those examined by RT-PCR had their quality and quantity confirmed using either a Bioanalyzer (Agilent) or Nanodrop (Nanodrop Co.). We performed one-color microarray analysis, and fluorescence-labelled (Cy3) cRNA probes were prepared from 400 ng of total RNA of each sample using a Low RNA Input Linear Amplification Kit (Agilent Technologies). Labeled cRNA probes (600 ng each) were then hybridized and washed using the Gene Expression Hybridization Kit and Gene Expression Wash Pack Kit (Agilent Technologies), according to the manufacturer's instructions. After being washed, the arrays were scanned using an Agilent Microarray Scanner (Agilent Technologies), and Feature Extraction ver. 9.1 (Agilent Technologies) was used for image analysis and data extraction. The microarray data from each sample were imported into GeneSpring 7.3 (Agilent Technologies) for further data characterization. The GEO accession number is as GSE23981 http://www.ncbi.nlm.nih.gov/geo/query/acc.cgi?acc=GSE23981, and Platforms are GPL9136 and GPL10134.

### Real-time RT-PCR(QPCR)

The total RNA was isolated from the fetal and endometrial tissues, BT-1 and SKIN cells, respectively used for microarray or RT-PCR analysis using TRIzol Reagent (Invitrogen, Carlsbad, CA, USA), according to the manufacturer's instructions. After verifying the quality of the RNA, one μg of total RNA was used for reverse transcription. The total RNA was then reverse transcribed into cDNA for 120 min at 37°C by MultiScribeTM reverse transcriptase (Perkin-Elmer Applied Biosystems, Foster City, CA) with a random primer dNTP mixture, MgCl2, and an RNase inhibitor.

The gene expression levels of fibroblast growth factor 2 (FGF2), follistatin (FST), heparin-binding epidermal growth factor-like growth factor (HBEGF), inhibin alpha (INHA), activin beta A (INHβA), activin beta B (INHβB), and transforming growth factor beta 1 (TGFβ1) were quantitatively confirmed by real-time RT-PCR analysis (QPCR) using the SYBR Green assay. Primer pairs were designed using the Primer Express 3.0 software program (Applied Biosystems) and are listed in Table 2. The thermal cycling conditions included the initial incubation of the samples at 50°C for two minutes and 95°C for ten minutes, followed by forty cycles of 95°C for fifteen seconds and 60°C for one minute. The cycle-threshold values (C_T_) indicate the quantity of the target gene in each sample. The relative difference in the initial amount of each mRNA species (or cDNA) was determined by comparing the CT values. The standard curves for each gene were generated by serially diluting plasmids containing FGF2, FST, HBEGF, INHA, INHβA, INHβB, TGFβ1, or GAPDH cDNA to quantify the mRNA concentrations produced. The expression ratio of each gene to GAPDH mRNA was calculated to adjust for any variation in the RT-PCR reaction. All values are presented as the mean ± SD. The expression of the inhibin βA gene during gestation was examined by another QPCR procedure. Briefly, one microgram of total RNA was reverse transcribed into cDNA in a total volume of 20 μL. QPCR was performed using SYBR Green fluorescence (Applied Biosystems, Foster City, CA, USA) to quantify inhibin βA gene expression. Bovine glyceraldehyde-3-phosphate dehydrogenase (GAPDH) was used as endogenous reference gene. The reaction mixture contained the cDNA template (1 μL), 0.4 μM each primer, SYBR Green PCR master mix (Applied Biosystems), and nuclease free water. The thermal cycling conditions included 2 min at 50 C and 10 min at 95 C. Thermal cycling proceeded with 40 cycles of 95 C for 15 sec and 60 C for 1 min. The primers were designed using the primer express software version 1.0, according to the manufacturer's instructions (Applied Biosystems). The primer sequences are given below together with their respective GenBank accession numbers within brackets. GAPDH (U85042): forward, 5'-aaggccatcaccatcttcca-3'; reverse, 5'-ccactacatactcagcaccagcat-3'. The primers were commercially synthesized (Espec Oligo Service, Japan), and QPCR detection was performed using an ABI PRISM 7700 sequence detector and SDS software version 1.7.

**Table 2:** Sequences of the oligonucleotide primers used for real-time RT-PCR

Gene name	Strand	Sequence
FGF2	Forward	5'-CCAGTTGGTATGTGGCACTGA-3'
	Reverse	5'-GGTCCTGTTTTGGGTCCAAGT-3'
FST	Forward	5'-GCGTGCTGCTGGAAGTGAA-3'
	Reverse	5'-CGGTGTCTTCCGAAATGGA-3'
HBEGF	Forward	5'-AGCTCCGGGTTCCAACCT-3'
	Reverse	5'-ATGGCACCTCTCTCCGTGAT-3'
INHA	Forward	5'-AGGAGGGCCTCTTCACGTATG-3'
	Reverse	5'-TCCAGTCCTGTGTGGAACCA-3'
INHβA	Forward	5'-GGCCACCCTCCCAAAGG-3'
	Reverse	5'-TTAACGGCCTCCACCATCTC-3'
INHβB	Forward	5'-CCGGGTCCGCCTGTACTT-3'
	Reverse	5'-GCCTGCACCACAAACAGGTT-3'
TGFβ1	Forward	5'-TGAGCCAGAGGCGGACTACT-3'
	Reverse	5'-TGCCGTATTCCACCATTAGCA-3'

### Western blot analysis

To analyze activin-like protein expression, BT-1 cell extracts (100 μg) and supernatants (200 μL) were applied to Western blotting, as described previously with some modifications [29]. In brief, these proteins were added to sample buffer and boiled for 3 min, before being applied to an SDS polyacrylamide slab gel according to the method of Laemmli [33] using a Mini Protean III apparatus (Millipore corporation, BioRad, MA, USA). The proteins were electroblotted onto a polyvinylidene difluoride membrane (Immobilon-P, Millipore) using transblot apparatus (transblot SD cell, BioRad). After being blocked with 5% ECL blocking agent (GE Healthcare, NJ, USA), the membrane was incubated with anti-activin βA diluted 1000-fold overnight at 4°C, followed by incubation with alkaline phosphatase-conjugated antibody (Sigma) for 1 h at room temperature. For the absorption experiment, 1 μg of primary antibody was incubated with 8 μg of purified activin A for 1 hr, before they were incubated with the membrane. Anti-activin βA antibody and purified activin A were prepared by Dr Hasegawa of Kitasato University [34]. Immunopositive bands were visualized using an Alkaline Phosphatase Conjugate Substrate Kit (BioRad).

### Statistical Analysis

The cell proliferation and QPCR data were analyzed by JMP software (SAS Institute Inc, Cary NC, USA) with one way ANOVA followed by the Dunnett's multiple comparison test. P-value of < 0.05 was considered significant.

## Results

### Gene expression levels of TGFβ super-family members in the fetal membrane and endometrium during implantation

**Figure 2:**
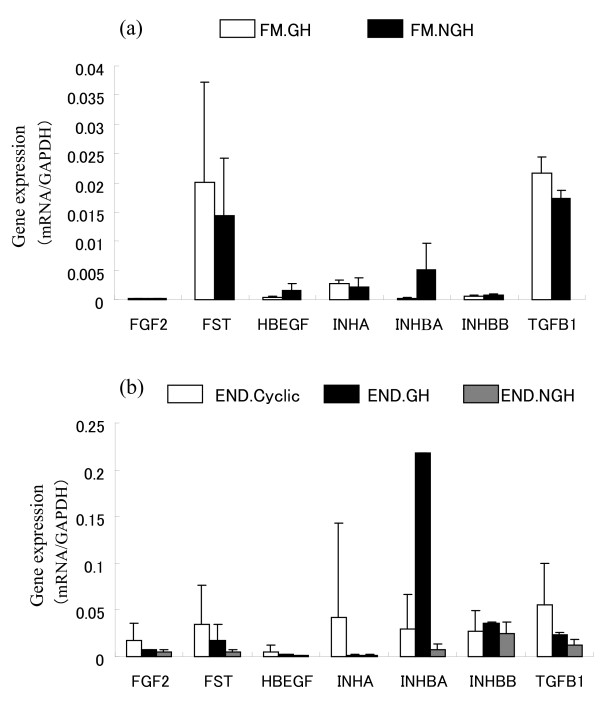
**Expression of activin and related genes in the fetal membrane on Day 35 of gestation**. The expression levels of FGF2, FST, HBEGF, INHA, INHBA, INHBB, and TGFB1 mRNA in gravid and non gravid horn tissues (FM.GH, FM.NGH, END.GH, and END.NGH) were detected by QRT-PCR. The expression of these genes was normalized to the expression of GAPDH measured in the same RNA preparation. The values are shown as the mean ± SD. FM.GH: gravid horn fetal membrane, FM.NGH: non-gravid horn fetal membrane, END.GH: gravid horn endometrium, END.NGH: non-gravid horn endometrium. END.Cyclic: endometrium in the luteal phase during the estrous cycle (day 7-14).

Around Day 35 of gestation, no placentomal villi (chorionic villi), which were identified as spots with fine capillaries, were found in the FM in the NGH, and less than 30 villi were macromorphologically confirmed in the FM in the GH (data not shown). Microarray analysis showed that the expression levels of various trophoblast specific genes were higher in the END of the GH than those in the END of the NGH; namely *CSH1*, PRP, PAG, AIF, and *Muc-1*. In FM, activin-related genes like INHA, *INHβA*, I*NHβB*, *FST*, *TGFβ1*, and *HB-EGF *were detectable in both the GH and the NGH; however, FGF2 was not detectable (Tables 3, 4). These data were confirmed by QPCR (Figure 2). There were no significant differences in the expression levels of these genes between the FM in the GH and NGH. No difference was seen in the endometrial *INHA *and *FST *expression levels between the GH and NGH, and quite high expression levels were detected during the estrous cycle compared to those observed during gestation although the difference was not significant. *INβA *expression in the GH.END was much higher than those in other tissues (over 0.2 vs. less than 0.05). In the GH, the expression levels of *INHβA*, *INHβB*, *FGF2*, and *IGF1 *in the END were significantly higher than those in the FM (p < 0.05).

**Table 3:** Gene expression in the fetal membrane on Day 35 of gestation

Gene	Gravid (n = 3)	Non-gravid (n = 2)	Ratio Gravid/Non-gravid
EGF	0.03 ± 0.03^a^	0.01	2.52
EGFR	1.68 ± 0.66	1.57	1.07
FGF2	0.01 ± 0.00	0.01	0.76
FST	84.74 ± 77.11	85.60	0.99
IGF1	0.02 ± 0.02	0.01	0.35
INHA	6.95 ± 2.37	6.10	1.14
INHBA	3.29 ± 2.84	248.39	0.01
INHBB	0.03 ± 0.03	0.15	0.19
TGFB1	1.41 ± 0.55	1.54	0.91
BMP3b	0.02	0.03	0.6
BMP4	1.41 ± 0.76	2.08	0.7
BMP15	0.01 ± 0.00	0.02	0.5
ACVR1	0.84 ± 0.13	1.02	0.8
ACVR2	0.90 ± 0.01	0.87	1.0
ACVR2B	0.48 ± 0.04	0.61	0.8
TGFBR1	1.02 ± 0.75	0.24	4.3
BMPR1A	0.10 ± 0.01	0.17	0.6
BMPR1B	0.04 ± 0.03	0.20	0.2
BMPR2	0.33 ± 0.13	0.37	0.9
SMAD2	7.30 ± 0.90	8.05	0.9
CSH1	184.28 ± 16.47	2.71	68.0
PAG1	72.83 ± 15.22	0.59	124.0
PRP1	13.55 ± 4.70	0.37	37.0
IFNT	0.04 ± 0.05	0.24	0.2

^a^Mean ± SD

**Table 4:** Gene expression in the endometrium on Day 35 of gestation

Gene	Gravid (n = 3)	Non-gravid (n = 3)	Ratio Gravid/Non-gravid
EGF	0.60 ± 0.95^a^	0.06 ± 0.04	10.58
EGFR	0.60 ± 0.35	0.78 ± 0.41	0.76
FGF2	0.05 ± 0.07	0.13 ± 0.01	0.39
FST	21.57 ± 10.07	11.35 ± 6.47	1.90
IGF1	0.07 ± 0.09	0.17 ± 0.20	0.40
INHA	0.96 ± 0.32	1.01 ± 0.48	0.96
INHBA	536.20 ± 314.75	31.78 ± 40.56	16.87
INHBB	0.14 ± 0.11	0.03 ± 0.01	5.55
TGFB1	0.51 ± 0.31	0.76 ± 0.26	0.67
BMP3b	0.01	0.01	1.0
BMP4	0.98 ± 0.49	0.87 ± 0.34	1.1
BMP15	0.15 ± 0.24	0.08 ± 0.11	2.0
ACVR1	0.83 ± 0.28	1.02 ± 0.14	0.8
ACVR2	0.13 ± 0.10	0.15 ± 0.01	0.8
ACVR2B	0.31 ± 0.16	0.42 ± 0.20	0.7
TGFBR1	0.13 ± 0.10	0.08 ± 0.07	1.6
BMPR1A	0.08 ± 0.07	0.32 ± 0.14	0.3
BMPR1B	0.01 ± 0.00	0.01 ± 0.00	1.0
BMPR2	0.29 ± 0.15	0.27 ± 0.09	1.1
SMAD2	4.93 ± 1.98	4.34 ± 0.65	1.1

^a^Mean ± SD

**Figure 3:**
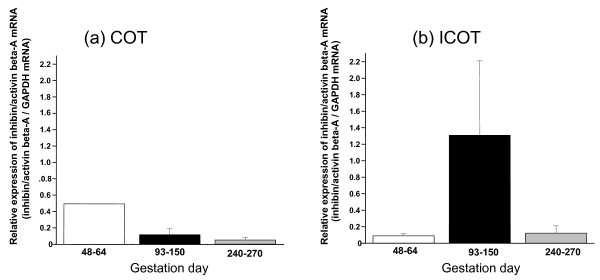
**Activin βA mRNA expression in cotyledonary (a, COT) and intercotyledonary (b, ICOT) tissues throughout gestation**. Gene expression was detected by QPCR.

**Figure 4:**
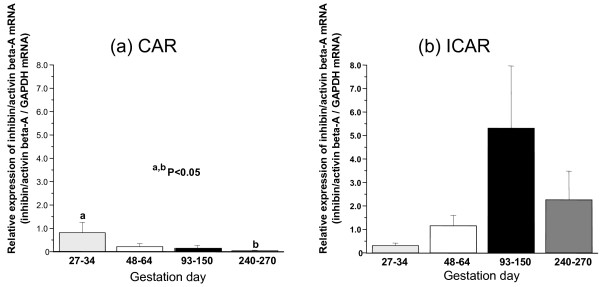
**Activin βA mRNA expression in caruncular (a, CAR) and intercaruncular (b, ICAR) tissues throughout gestation**. Gene expression was detected by QPCR.

When *INHβA *expression throughout gestation was analyzed by QPCR, higher expression levels were detected in the COT during early gestation and in the ICOT during the middle period of gestation. Similar expression profiles were confirmed in the CAR and ICAR. However, two to three times higher expression intensities were detected in area from the END to the FM (Figures 3, 4).

### Expression of TGFβ super-family members in BT-1 cells

**Figure 5:**
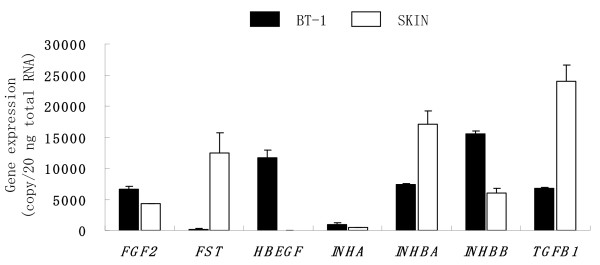
**Expression of activin and related genes in trophoblastic cells (BT-1)**. Gene expression was detected by QPCR in BT-1 and SKIN cells. The expression of these genes is shown as copy number per 20 ng total RNA. The values are shown as mean ± SD. BT-1: a trophoblastic cell line established by Shimada et al. (2001), SKIN: cells derived from bovine skin as a control.

BT-1 cells possess some trophoblastic cell features derived from in vitro fertilized ova [30], so we used them as a representative source replacing in vivo trophoblastic cells from the extra-embryonic membrane. First, the expression of various growth factors/cytokines was examined by QPCR. As a result, BT-1 cells were found to express *FGF2*, *HBEGF*, *INHβA*, *INHβB*, *FST*, and *TGFβ1*, all of which were also detected in SKIN cells, which were used as the control (general somatic cells) (Figure 5). There were significant differences between the expression levels of these molecules in BT-1 and SKIN cells (except for in *INHA *expression) (P < 0.05).

### Effects of trophoblast extracts on cell proliferation

**Figure 6:**
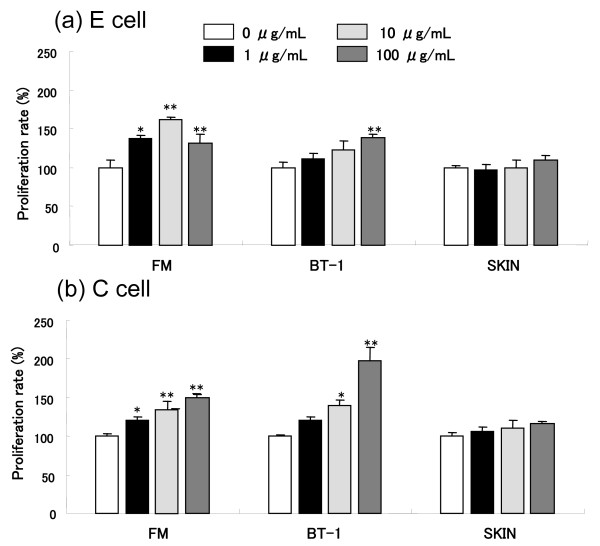
**Effects of trophoblastic tissue extracts on the proliferation of fibroblast cells**. EM: trophoblast tissue extracts with protein concentrations ranging from 1~100 μg/mL, BT-1: cell extracts with the same protein concentrations as the EM, SKIN: cell extracts from the skin. These extracts were applied to endometrial fibroblast cells (E) and cotyledonary fibroblast cells (C). The values (mean ± SD) are expressed as percentages relative to the value obtained for the relevant untreated group (control). Dunnett's multiple comparison test was carried out to test the differences between the control and the treatment groups: *P < 0.05, **P < 0.01.

To examine whether trophoblastic factors stimulate endometrial cell proliferation, extracts containing 1-100 μg/mL proteins from the fetal membrane, BT-1, and SKIN cells were applied to in vitro cell cultures of two cell lines with different origins, E as bovine endometrial fibroblast cells and C as bovine cotyledonary fibroblast cells. The trophoblast and BT-1 extracts stimulated cell proliferation in both E and C cells (Figure 6a and 6b, respectively); however, the SKIN cell extract had no effect. Cell proliferation analysis using C cells showed that the extracts from trophoblasts and BT-1 cells enhanced cell growth.

### Activin protein expression in trophoblast cells

**Figure 7:**
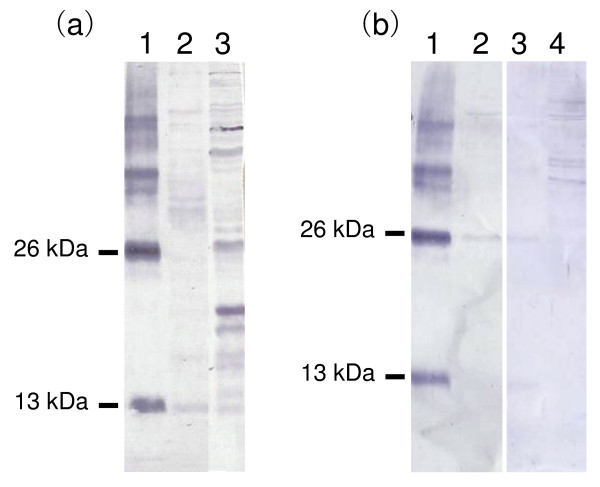
**Immunological activin βA analysis in trophoblast cells (BT-1)**. (a) Activin protein was detected by Western blotting using anti-activin βA sera. Lane 1: purified activin as a positive control, lane 2: culture medium of BT-1 cells, lane 3: BT-1 cell extract. (b) Activin protein was detected using anti-activin βA sera, which was absorbed with an excess of purified activin βA protein (Lane 3, 4). Lanes 1 and 3: purified activin A, lanes 2 and 4: BT-1 extracts.

We next examined whether activin protein is expressed in trophoblastic cells using BT-1 cells as a model. Although activin-βA protein was found in BT-1 extract and culture supernatant by Western blot analysis, only small amounts of the appropriate sized proteins were detected in BT-1 cells and culture media (Figure 7). The activin-βA subunit was found in BT-1 culture supernatant as a 13 kDa protein but many nonspecific bands including a 26 kDa protein were also found in the extract (Figure 7a). The excess application of semi-purified activin to anti-activin sera showed the simultaneous disappearance of both the 13 and 26 kDa proteins in BT-1 cells.

### Cell proliferative activity of growth factors

**Figure 8:**
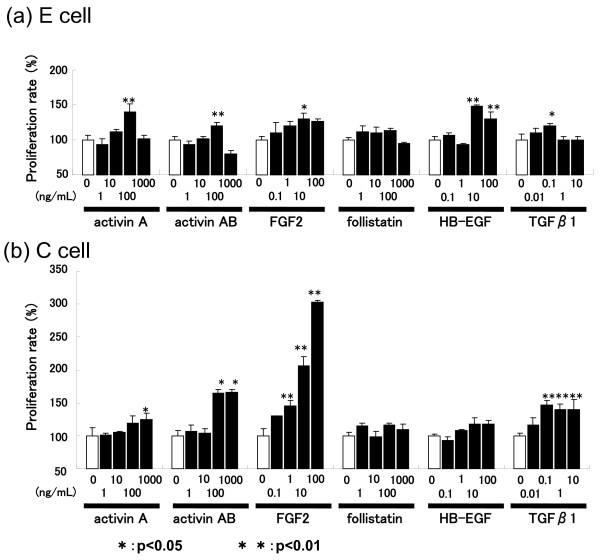
**Cell proliferation analysis of activin and related growth factors**. Purified activin A, activin AB, and follistatin were applied to E and C cells at concentrations ranging from 1 to 1000 ng/mL. FGF2 and HB-EGF were used at concentrations ranging from 0.1 to 100 ng/mL. TGFβ1 was used at concentrations ranging from 0.01 to 10 ng/mL. The values (mean ± SD) are expressed as percentages relative to the value obtained for the relevant untreated group (control). Dunnett's multiple comparison test was carried out to test the differences between the control and treatment groups: *P < 0.05, **P < 0.01.

To confirm whether activin displays proliferative activity in the endometrium, we applied various growth factors including activin-A and -AB to E and C fibroblast cell preparations. Although activin-A and -AB, FGF2, in and TGFβ stimulated the proliferation of both cell lines, follistatin had no effect on either cell type. HB-EGF stimulated E cell growth (Figure 8).

## Discussion

Implantation is a complex process involving various factors and biochemical and molecular modifications: cell-to-cell contact between cells with different origins, cell invasion and/or fusion depending on species, endometrial cell proliferation, and matrix reorganization. In the ruminant endometrium, feto-maternal events mainly occur in a limited area named the caruncle [1,35]. However, how and why cell-cell contact and active cell proliferation are initiated in the caruncle in ruminants rather than in the intercaruncle remain answered. During implantation, the embryo is stimulated by maternal factors, uterine milk protein and serpin [36,37], which ensure a suitable environment for fetal development. At the same time, endometrial functions, and especially those in the caruncle in bovines, are regulated by fetal-side factors, for example interferon-tau, which stimulates endometrial prostaglandins and the ovarian corpus luteum, which functions as a factor for the recognition of gestation [38-42]. In the present study, we explored the possible role of activin, a member of the TGFβ super-family, at the feto-maternal interface. As in other species such as humans and mice, activin plays a crucial role in endometrial remodelling, but there is little information regarding the role of the TGFβ super-family in ruminants [15,43]. Although the structure of the bovine placenta is different from those of humans and mice; namely, the former is a non-invasive placenta but the latter is an invasive placenta, endometrial remodelling is still necessary for placentogenesis in bovines [44]. Discordant feto-maternal organization causes embryo loss. The caruncular and intercaruncular area play different roles in the bovine endometrium, and their gene expression patterns have been identified [45,46]

Extracts from the extra-embryonic membrane stimulated cell proliferation in an in vitro fibroblast cell culture, and BT-1 cells, which are derived from bovine blastocysts and have trophoblastic features, also stimulated cell proliferation effectively (Figure 4). These data suggest that trophoblastic cells contain a factor that stimulates bovine endometrial cell proliferation in the caruncle. Although trophoblast tissue expresses many factors including vascular factors (VEGF and their receptors, TGF, IGF, PIGF, etc. [47]), the most important factor remains unknown. Microarray and QPCR confirmed that various molecules including *FGF2*, *INHβA*, *INHβB*, *FST*, *HBEGF*, and *TGFβ*. were detectable in the END; however, most of them were highly expressed in the END.GH. A comparison between the gene expression patterns in gravid and non-gravid horn trophoblasts showed that in the fetal membrane specific molecules such as placental lactogen (PL), prolactin-related protein (PRP), and pregnancy-associated glycoprotein (PAG) were expressed more in gravid horn trophoblasts. These results agreed with those of a previous report [8] and imply that members of the TGFβ super-family including activin play roles in endometrial receptivity in ruminants. As in the gravid horn, the fusion of trophoblast cells and the endometrial epithelium starts earlier than in the non-gravid horn; namely, it may be evidence of the initiation of implantation [8,43].

Activin was detected in bovine blastocysts as well as in human and mouse blastocysts [11,13,48,49]. It stimulates the development of the embryo; i.e., cell proliferation of the blastocyst and attachment in humans and mice [48,50,51]. Placental villi produce activin A and promote hormone production, and these hormones direct the formation of the trophoblastic syncytium [52]. Although endometrial activin production is known to occur in these species [26], there is no information regarding its function in the bovine caruncular endometrium. TGFβ super-family genes including activin genes (*INHβA *and *INHβB*) may play crucial roles in the proliferation of various cell types such as endometrial, immune, stem, ovarian and tumor cells [53-55]. In the present study, high *INHβA *expression was found in the gravid endometrium, and our findings suggest that activin plays a similar role as a regulator of endometrial remodelling in humans even though bovine endometrial cells are not involved in decidualization during implantation [17,52]. Endometrial remodelling is necessary for bovine implantation [56,57], and a comparison between gravid and non-gravid endometrial molecular expression implied that the initiation of implantation and cell proliferation in the bovine caruncular endometrium is regulated by fetal-side trophoblasts. However, there are no reports regarding fetal-side stimulation. We hypothesized that trophoblastic factors participate in rapid spatiotemporal endometrial cell proliferation during bovine implantation. Using BT-1 cells, which possess trophoblastic features, we attempted to identify activin and detected various molecules immunologically (Figure 5). Extracts from fetal trophoblastic tissues and BT-1 cells stimulated cell proliferation. These findings suggest that fetal-side tissues stimulate endometrial remodelling and development during implantation. Activin activity is regulated by various factors including FST, and FST expression is higher than that of activin genes in bovine trophoblastic tissues [20,55]. Therefore, activin may not be active in fetal trophoblasts at this time. Although activin is not the only factor that stimulates cell proliferation in the bovine endometrium, it may play an important role in endometrial remodelling and development [52]. The detailed roles of activin in bovine endometrial remodelling and implantation require further study.

In the present study, other molecules were found to be expressed in the fetal and endometrial tissues, (FGF2, HB-EGF, and TGFβ1), but their biological roles are still obscure. Almost no expression of FGF2 was detected in the trophoblastic tissues but it did demonstrate proliferative activity in cell culture. Endometrial FGF2 may be important for endometrial remodelling and implantation and may be related to interferon tau expression [58,59]. In addition, HB-EGF may play a specific role during implantation on both the maternal and fetal sides [12,60,61]; however, this requires further study.

## Conclusions

TGFβ super-family genes are specifically expressed in the bovine endometrial and trophoblastic tissues in a specific spacio-temporal manner. *INHβA *(activin βA) was found to be highly expressed in the gravid horn endometrium, but its expression was low in trophoblasts. Several factors derived from trophoblastic tissues stimulated the proliferation of fibroblast cells, and activin may participate in endometrial proliferation. These results suggest that TGFβ super-family members including activin, which are produced by trophoblasts and the endometrium, play a crucial role in endometrial remodeling during the peri-implantation period in cattle. In addition, bovine trophoblastic tissues contain various other cytokine/growth factors that may be important for placental growth during bovine placentation.

## Competing interests

The authors declare that they have no competing interests.

## Authors' contributions

KS and KK participated in the design of study, carried out the experiments and prepared the manuscript. CBH performed QPCR for TGFβ super-family during gestation. YH purified Activn, Inhibin and raised their antibody. KH participated in the design and coordination of study and helped to draft the manuscript. All authors read and approved the final manuscript for publication.
